# Supplementary material for: Using mixed-effects modeling to estimate decay kinetics of response to SARS-CoV-2 infection
Source: Antib Ther. 2021 Jun 25;4(3):144–8. doi: 10.1093/abt/tbab013 (PMC8287637; doi:10.1093/abt/tbab013)
Supplement: Bottino_et_al_SupplementaryInformation_v2_tbab013 [file bottino_et_al_supplementaryinformation_v2_tbab013.docx]

# Supplementary materials for Bottino et al, Antibody Therapeutics (2021)

## Supplementary methods

To isolate the effect of using mixed effects modeling versus naïve-pooled parameter estimation (rather than embarking on a model-building activity), we used the default Monolix (2020R1, Lixoft) settings and the same single-exponential model used by (Dan 2021) to fit PSV observations ($y_{obs}(t)$):

$$y_{obs}(t)=y_{0}e^{{-k}_{el}t}[1+b\epsilon]$$

The parameter $y_{0}$ is the estimated PSV titer at symptom onset,$e$ is Euler’s number, $k_{el}$ is the PSV titer exponential elimination rate (1/day), $t$ is time in days post-symptom onset, $b$ is the estimated magnitude of proportional error between the model prediction and noisy observations, and $\epsilon$ is a standard normally distributed random variable.

We performed nonlinear mixed effects modeling using Monolix Suite 2020R1 (Lixoft). By default, monolix uses interval censoring information (in this case BLOQ PSVNT between 0 and 20) to properly integrate the likelihood function over the interval (often referred to as the ‘M3’ method in MEM community – Beal 2001). The MEM-formatted dataset were derived from the dataset provided in Supplementary Materials from (1); the Monolix project and model files, input dataset and R code for generating the figures are included in the supplementary materials.

By the standard MEM framework, we assumed inter-individual variability on both model parameters ($y_{0}$ and $k_{el}$), which were both assumed to be lognormally distributed across the population. In other words, the parameters $y_{0}^{j}$ and $k_{el}^{j}$ for the $j^{th}$ individual are given by:

$$y_{0}^{j}=\theta_{y_{0}}e^{\eta_{y_{0}}^{j}}$$

$$k_{el}^{j}=\theta_{k_{el}}e^{\eta_{k_{el}}^{j}}$$

The parameters $\theta_{y_{0}}$ and $\theta_{k_{el}}$ are the estimated ‘typical values’ of $y_{0}$ and $k_{el}$ for the population, and $\eta_{y_{0}}^{j}$ and $\eta_{k_{el}}^{j}$ are the estimated perturbations to $\theta_{y_{0}}$ and $\theta_{k_{el}}$ giving the maximum likelihood estimates of $y_{0}$ and $k_{el}$ for individual $j$. These perturbations $\eta_{y_{0}}^{j}$ and $\eta_{k_{el}}^{j}$ are assumed to come from a normal distribution centered at 0 with estimated standard deviations $\omega_{y_{0}}$ and $\omega_{k_{el}}$ respectively. Given an individual-level longitudinal population dataset, such as the PSV neutralizing titers for the 41 patients having PSVNT values for at least two distinct times (Fig S1), and initial parameter guesses chosen interactively in the MEM software (e.g., MONOLIX 2020R1) to roughly describe trends in the data, the MEM software simultaneously provides estimates for the population parameters $\theta_{y_{0}}$ and $\theta_{k_{el}}$ , and their inter-individual variability standard deviations $\omega_{y_{0}}$ and $\omega_{k_{el}}$, proportional error magnitude $b$, and a set of ‘random effects’ for each individual: ${\{\eta}_{y_{0}}^{j}\},{\{\eta}_{k_{el}}^{j}$}. The population-level parameter estimates are given in Table S1. To account for uncertainty in the individual parameter estimates for $y_{0}$ and $k_{el}$ given the population estimates and the sparse individual data, we estimated 1000 parameter sets from the posterior parameter distribution of each patient. We then reported the histogram of the 41000 estimates for half-life ($\tau_{1/2}=\frac{\ln2}{k_{el}}$) in Fig 1.

We also performed a Visual Predictive Check (VPC), a graphical tool that assesses how well a MEM captures both the central tendency as well as the population variability in the observed data. Figure S2 shows the VPC for the single exponential model, which we determined to be adequate.

To estimate the loss of sensitivity across the population over time, we simply calculated the time for each patient to decay to the limit of sensitivity $LOS$ reported in Dan et al (LOS=20 for PSVNT): $t_{j}^{*}=-\frac{1}{k_{el}^{j}}\ln\frac{LOS}{y_{0}^{j}}$. To test our prediction of loss of sensitivity over time, we used the R (3.5.1) ‘survival’ package (2.42-3) in RStudio (1.1.456, RStudio, Inc) to generate the empirical distribution of times as well as 90% bootstrapped confidence intervals and overlaid it on the model predictions (Fig 2).

We additionally tested the (Wheatley 2021) data set, for which a bi-exponential model was reported, and found that the Wheatley data did support a bi-exponential model by our methodology, while the single-exponential model was selected over the bi-exponential model for the Dan et al data set (See supplementary section: “To Test the Bi-exponential model…”).

## Supplementary Figures and Tables

|  | **Parameter description** | **Typical value** $\boldsymbol{(\theta)}$ | **RSE%** | **Population SD** $\boldsymbol{(\omega)}$ | **RSE%** |
| --- | --- | --- | --- | --- | --- |
| $y_{0}$ | PSV neutralizing titer at symptom onset | 228.7 | 12.2 | 1.24 | 6.87 |
| $k_{el}$ | PSV neutralizing titer elimination rate (1/day) | 0.0067 | 11.1 | 0.58 | 15.8 |
| b | Proportional error scale for PSV | 0.41 | 9.54 |  |  |

**Table S1:** Population parameter estimates from MEM. RSE = Relative Standard Error. Population standard deviations (SD) are in lognormal space as detailed in the methods; a population SD of 1 corresponds to ~100% variability in the population for that parameter. The RSE values approximate uncertainty in the population parameter estimates (not to be confused with inter-individual variability, which is given by the $\omega$ values).

| Monolix model file🡪 | M01_single_exponential.txt | M04_piecewise_2_decay.txt |
| --- | --- | --- |
| Data set | $y(t)=y_{0}e^{{-k}_{el}t}$ | $y(t)=\left\{ \begin{matrix} y_{0}e^{{-k}_{1}^{el}t} & , t\leq T_{0} \\ y\left( T_{0} \right)e^{-k_{el}^{2}\left( t-T_{0} \right)} & ,t>T_{0} \end{matrix} \right.$ |
| Dan dataset (N=41) | **1184.27** | 1185.92 |
| Wheatley dataset (N=64) | 3216.14 | **3154.02** |

**Table S2:** Comparison of single exponential (M01) and piecewise 2-exponential (M04) models on the Dan and Wheatley data sets. The numbers in the table are the Importance Sampling approximated Corrected Bayes Information Criteria (BICc) scores. As BICc penalizes for additional complexity, the better model in each row is the one with the lower BICc score (bold). Note that the models can be nested (making BICc comparison appropriate) by setting T_0_ sufficiently large. We believe the more complex model was selected for the Wheatley data set because more of those patients had at least 3 time points compared to the Dan data set.

| **PSV neutralizing titer** | 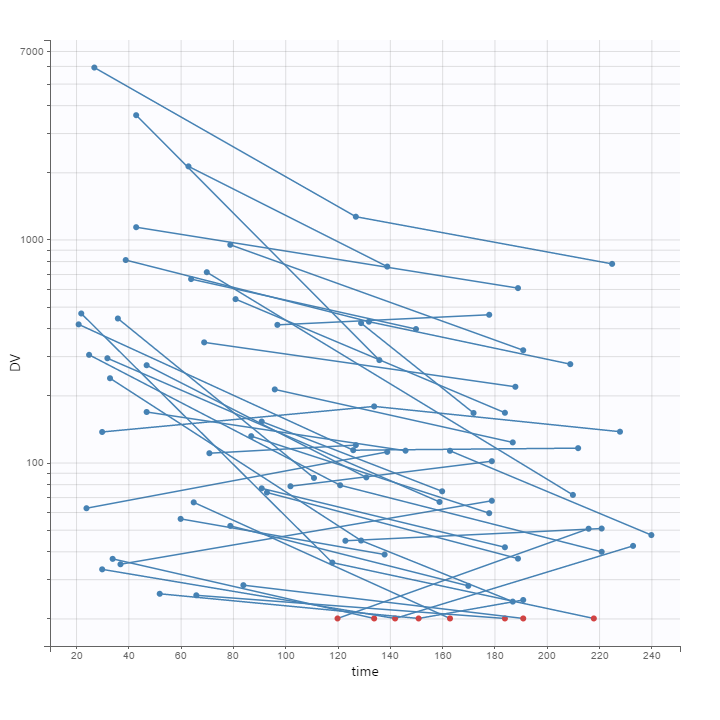 |
| --- | --- |
|  | **Days post symptom onset** |

**Figure S1**: PSV neutralizing titer (PSVNT) data used in the analysis. Below lower limit of quantification data (LLOQ = 20) are shown as red dots. Data points from the same patient are joined with a blue line. Time is in days. Only the 41 patients with at least two distinct time points for PSVNT were included in the MEM analysis. (raw data in supplementary files – Below limit of quantification (BLOQ) data is indicated by the CENS column in the raw data set, which triggers Monolix to use the M3 method (Beal))

| PSV neutralizing titer | 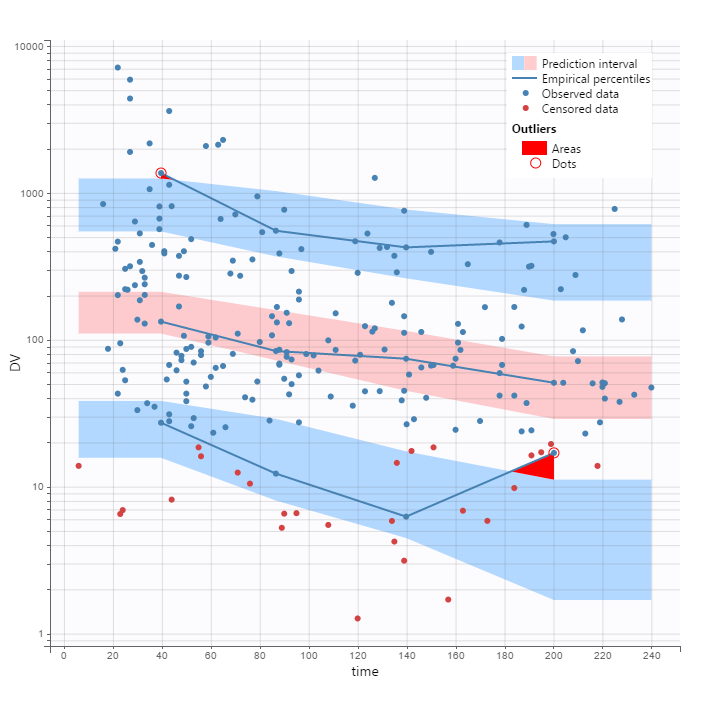 |
| --- | --- |
|  | Days post symptom onset |

**Figure S2**: Visual Predictive Check (VPC) for Pseudovirus Neutralizing Titer (PSV). The lower blue, middle pink, and upper blue bands represent the model-predicted 5, 50 and 95% population quantiles of the observations, while the blue lines represent the percentiles observed across the raw data. The red areas highlight deviation between the predicted and observed percentiles. The blue dots represent the observed data, and the red dots represent the simulated values of below limit of quantification (BLOQ) observations. The BLOQ values are simulated to avoid truncation artifacts in the VPC.

## Reproducing the figures and tables (requires copy of Monolix software):

1. Copy entire Supplementary Materials directory, preserving directory structure. (This may be found at <https://github.com/deanbot1/immunomem>, or alternatively the files may be obtained from the Supplementary Materials online. We recommend starting from the GitHub location.)
2. Launch monolix (2020R1) and load project: *monolix/run015_PSV-neut-titer_M01-base.mlxtran*
3. Run the full project per monolix instructions
   1. Observe **table 1** in population results
   2. Observe **figure S1** in data plot tab
   3. Observe **figure S2** in Visual Predictive Check tab
4. Launch R (Rstudio), install required packages as needed.
5. Make sure working directory (setwd()) is *code/*
6. Source *postprocess_monolix.R* to generate **figure 1**
7. Source *postprocess_monolix_bootstrap+PPC.R* to generate **figure 2**

## To test the bi-exponential model and mono-exponential model on the Dan and Wheatley data sets (requires copy of Monolix software):

1. For the Wheatley data set:
   1. Launch monolix and load project: *M01_Wheatley1b_base.mlxtran*
   2. Run the project and note the BICc score W1. This is single exponential model (M01).
   3. Load the project: *M04_Wheatley1b_base.mlxtran*
   4. Run the project and note the BICc score W2. This is the piecewise 2-exponential model modeled after Wheatley’s report of 2 exponentials (models/M04).
   5. Note that W2 < W1, indicating that Bayes Information Criteria favors the 2 exponential model for this data set.
2. For the Dan data set
   1. Launch monolix and load project: *M01_Dan_PSV-base.mlxtran*
   2. Run the project and note the BICc score D1. This is single exponential model
   3. Load the project: *M04_Dan_PSV-base.mlxtran*
   4. Run the project and note the BICc score D2. This is the 2 exponential model M04.
   5. Note that D2 > D1, indicating that BIC favors the 1-exponential model for the Dan data set.

See also table S2.

## Supplementary references

Beal SL. Ways to fit a PK model with some data below the quantification limit. *J Pharmacokinet Pharmacodyn.*2001;28(5):481–504. doi: 10.1023/A:1012299115260.

Dan et al, Science, Immunological memory to SARS-CoV-2 assessed for up to 8 months after infection, 2021.

Wheatley et al, Nature, Evolution of immune responses to SARS-CoV-2 in mild-moderate COVID-19, 2021.
